# Poly(1-vinylimidazole) polyplexes as novel therapeutic gene carriers for lung cancer therapy

**DOI:** 10.3762/bjnano.11.26

**Published:** 2020-02-17

**Authors:** Gayathri Kandasamy, Elena N Danilovtseva, Vadim V Annenkov, Uma Maheswari Krishnan

**Affiliations:** 1 Centre for Nanotechnology & Advanced Biomaterials (CeNTAB), School of Chemical and Biotechnology, SASTRA Deemed University, Thanjavur – 613401, Tamil Nadu, India; 2 Limnological Institute of the Siberian Branch of the Russian Academy of Sciences, 3, Ulan-Batorskaya St., P.O. Box 278, Irkutsk, 664033, Russia

**Keywords:** anti-VEGF siRNA, gene silencing, lung cancer, microarray, poly(1-vinylimidazole), small interfering RNA (siRNA), vascular endothelial growth factor (VEGF)

## Abstract

The present work explores the ability of poly(1-vinylimidazole) (PVI) to complex small interfering RNA (siRNA) silencing vascular endothelial growth factor (VEGF) and the in vitro efficiency of the formed complexes in A549 lung cancer cells. The polyplex formed was found to exhibit 66% complexation efficiency. The complexation was confirmed by gel retardation assays, FTIR and thermal analysis. The blank PVI polymer was not toxic to cells. The polyplex was found to exhibit excellent internalization and escaped the endosome effectively. The polyplex was more effective than free siRNA in silencing VEGF in lung cancer cells. The silencing of VEGF was quantified using Western blot and was also reflected in the depletion of HIF-1α levels in the cells treated with the polyplex. VEGF silencing by the polyplex was found to augment the cytotoxic effects of the chemotherapeutic agent 5-fluorouracil. Microarray analysis of the mRNA isolated from cells treated with free siRNA and the polyplex reveal that the VEGF silencing by the polyplex also altered the expression levels of several other genes that have been connected to the proliferation and invasion of lung cancer cells. These results indicate that the PVI complexes can be an effective agent to counter lung cancer.

## Introduction

Gene therapy is a promising strategy that can be employed in the treatment of many hereditary disorders as well as diseases triggered by sporadic mutations including many forms of cancer. However, the therapeutic potential of gene therapy is yet to be realized completely due to the challenges associated with stability, target specificity and transfection [1]. The use of viral or non-viral vectors to deliver the therapeutic oligonucleotide to the target cell has been widely explored to overcome the inherent problems associated with the administration of the naked oligonucleotide [2]. The majority of gene delivery studies have employed viral vectors due to their superior transfection capabilities. But the high frequency of mutations and packing limitations associated with viral vectors necessitate the search for safer alternatives [3]. In this context, non-viral vectors have garnered interest in recent years as gene delivery vehicles. But the highly cationic nature of the employed carriers is associated with immunogenicity and toxicity. Further, the ability of these carriers to escape the acidic endosomes in the cells limit their transfection efficiency [3]. One of the widely explored non-viral polymeric carriers for gene delivery is poly(ethylene imine) (PEI), which can effectively escape from the endosomes through the “proton sponge” mechanism [4]. However, PEI systems are limited by their toxicity and, hence, there is a need for less toxic but still effective gene carriers.

The imidazole ring is involved in many physiological processes and therefore imidazole-containing systems have been investigated for a myriad of biological applications [5]. Poly(vinylimidazole) is a water-soluble polymer, which has been synthesized as poly(1-vinylimidazole) and poly(4-vinylimidazole) [6] by using different methods. The imidazole groups can be protonated at acidic pH values conferring cationic character to the polymer. The conformation of PVI chains were found to be altered on protonation that is dependent on the anions present in the system [7]. Several studies have explored the potential biomedical applications of PVI and its derivatives. Pullulan-grafted poly(1-vinylimidazole) was found complex anionic citrate and tripolyphosphate effectively in acidic medium via the imidazole moiety [8]. The catalytic properties of poly(1-vinylimidazole) due to the proton-donating nature of the imidazole moiety have been demonstrated in [9]. Imidazole-based hydrogel films demonstrated excellent anti-microbial effects [10]. A copolymer of poly(acrylamide) and poly(vinylimidazole) was used as a hydrophilic matrix to disperse multiwalled carbon nanotubes and the enzyme glucose oxidase for glucose-sensing applications [11]. A hydrogel of xanthan gum and poly(1-vinylimidazole) was recently explored for protein encapsulation and delivery. The system exerted no toxic effects on cells and maintained the functionality of the protein [12]. A pyrrole–imidazole polyamide system was found to inhibit prostate cancer progression through interfering with the expression and function of the androgen receptor [13]. Chitosan–imidazole derivatives have been also explored for gene transfection in HEK293 cells [14].

In recent years, poly(vinylimidazole)-based systems have emerged as a front-runner for gene delivery applications due to their polycationic nature, biocompatibility as well as the ability to escape the endosome by activating the proton sponge mechanism. In an earlier report, histidylated poly(ʟ-lysine) was found to exhibit high transfection efficiency enabled by a pH-responsive endo-lysosomal escape [15]. It was therefore expected that poly(vinylimidazole) side chains will show a higher transfection efficiency. Alkylated poly(1-vinylimidazole) with different chain lengths has been investigated for DNA complexation and transfection in HepG2 liver cancer cells. Butylated PVI was found to be nontoxic and the most effective when compared to other alkyl derivatives regarding DNA complexation [16]. Carboxymethyl poly(1-vinylimidazole) has also been investigated for DNA complexation and was found to exert no toxicity to cells [17]. Poly(1-vinylimidazole) chains modified with aminoethyl groups demonstrated excellent DNA binding ability in synergy with lactosylated poly(ʟ-lysine). This system was found to exhibit excellent gene transfection ability specifically in hepatocytes through interactions with the asialoglycoprotein receptor expressed on the hepatocyte surface through the lactosylated poly(ʟ-lysine). The endosomal escape was mediated through a pH-responsive protonation of the imidazole and amino moieties that disrupted the endosomal membrane [17]. Zinc–PVI systems have also been investigated for complexing DNA for gene delivery applications [18].

A folic acid-conjugated amine containing poly(1-vinylimidazole) was found to effectively complex DNA and transfect cancer cells [19]. PVI linked with the dipeptide Cys–Trp was demonstrated to self-assemble to micelles that could also complex RNA effectively. Very few studies have also attempted to investigate the DNA complexation efficiency of poly(4-vinylimidazole) polymers that also possesses low cytotoxicity and good endosomal escape properties [20].

To our knowledge, only few proof-of-concept studies have been carried out to explore the potential of PVI as a cyto-compatible gene carrier. The present work aims to synthesize poly(1-vinylimidazole) for the delivery of anti-VEGF siRNA to lung cancer cells and explore for the first time the effect of VEGF silencing on differential expression of genes and on cell viability, migration and chemosensitization.

## Experimental

### Materials

PVI was obtained via the polymerization of a 30% ethanolic solution of 1-vinylimidazole in the presence of 2% (by monomer mass) of 2,2-azobis(isobutyronitrile) in argon atmosphere at 60 °C by Prof. Annenkov’s group and the polymer fraction with a molecular weight of 35,000 Da was used for the study. Detailed synthesis and characterization of the polymer was earlier reported by Prof Annenkov [21]. The lung cancer cell line A549 was procured from the National Centre for Cell Sciences (NCCS), Pune. Human umbilical vein endothelial cells (HUVECs) were procured from ATCC, USA. EGM^TM^ endothelial cell growth medium BulletKit^TM^ was procured from Lonza, USA. The VEGF siRNA sequence (sense 5′-GGA-GUA-CCC-UGA-UGA-GAU-CTT-3′, antisense 5′-GAU-CUC-AUC-AGG-GUA-CUC-CTT-3′) and cyanine-3 fluorescent tagged VEGF siRNA (sense: 5′-CY3-GGA-GUA-CCC-UGA-UGA-GAU-CTT-3′, antisense: 5′-CY3-GAU-CUC-AUC-AGG-GUA-CUC-CTT-3′) with λ_ex_ of 550 nm and λ_em_ of 570 nm were purchased from Eurofins Genomics, USA. Scrambled siRNA (sense 5′-ACG-UGA-CAC-GUU-CGG-AGA-A55-3′, antisense: 5′-UUC-UCC-GAA-CGU-GUC-ACG-U55-3′) procured from Eurogentech, USA was used for comparison in the study. Migration transwell inserts (8 µm) pore size were procured from HiMedia, USA. Ribogreen reagent was purchased from Invitrogen, USA. All other reagents of analytical grade were purchased from Merck, India. 5-Fluorouracil (5-FU) was procured from Sigma-Aldrich, USA. VEGF antibody (Santa Cruz Biotechnology Ltd., USA), β- actin and other antibodies (Cell Signaling Technology, USA) were used in the study. Microarray consumables were purchased from Cell Signaling Technology, USA. RNAse-free water was used for preparation of buffers and all solutions.

### Methods

#### Preparation of the PVI–siRNA polyplex

A stock solution of PVI (100 mg/mL) was prepared using RNAse-free water. The pH value was maintained at 7.0. A siRNA stock solution (10 µM) was prepared using RNAse-free water. For complexation, the aqueous solutions of PVI and siRNA were mixed at different ratios (v/v). The resultant mixtures were vortexed followed by incubation for 30 min at room temperature.

#### Characterization of the polyplex

**Electron microscopy:** A small drop of the polyplex sample was placed on a conducting carbon tape and air-dried. The sample was then sputter-coated with a thin film of gold. The sample was placed in the sample chamber and imaged at an accelerating voltage of 3 kV using a cold field-emission scanning electron microscope (JSM6701F, JEOL, Japan). For transmission electron microscopy, 1 mL of the polyplex was deposited on 400 mesh copper grids (Canemco-Marivac, Canada) and air-dried for 10 min. The excess sample was removed by blotting using a filter paper. The grids were washed using RNAse-free water and dried overnight. The samples were stained with 1% phosphotungstic acid solution (Merck, Germany) and imaged using high-resolution field-emission transmission electron microscopy (JEM2100F, JEOL, Japan).

**Differential scanning calorimetry:** The melting point of the blank PVI nanoparticles and of the PVI–siRNA polyplex were recorded using differential scanning calorimetry (DSC, Polyma 214, Netzsch, Germany) in the temperature range of 0–300 °C in a nitrogen atmosphere at a scan rate of 5 °C·min^−1^. The samples were placed in an aluminium pan with lid, which also served as the reference.

**Fourier-transform infrared spectroscopy (FTIR):** The FTIR spectra of the free siRNA, blank polymer nanoparticles and the polyplex were recorded between 4000 and 400 cm^−1^ averaging 10 scans per run in attenuated total reflection mode (ATR) using a Fourier-transform infrared spectrometer (Spectrum 100, Perkin Elmer, USA).

**Dynamic light scattering (DLS) and zeta potential measurements:** The hydrodynamic size of the PVI–siRNA polyplexes was measured on a Zetasizer Nano ZS (Malvern instruments, UK). Samples at different volume ratios containing a final siRNA concentration of 100 nM were used for the measurements. The measurements were made using a quartz microcell of 1 mL capacity and 3 mm path length. Clear disposable cells were employed for zeta potential measurements. All measurements were performed for each sample in triplicates 20 min after sample preparation at 25 °C.

**Electrophoresis:** The polyplexes with a tracking dye (bromophenol blue, 2 µL), were loaded on a 1% agarose gel. The electrophoresis was carried in Tris/boric acid buffer (TBE) buffer at 80 V for 45 min. The gel was imaged after staining with ethidium bromide in a UV transilluminator using a gel documentation system (Fusion SoloX, Vilber Lourmat, France).

**Heparin displacement assay:** Polyplexes were prepared in a volume ratio of 4:1 with a final siRNA concentration of 100 nM and incubated with heparin (low molecular weight fraction, Sigma-Aldrich, USA) solutions of different concentrations for 30 min and expressed as heparin/siRNA (v/v) ratio. The samples were electrophoresed on a 1% agarose gel containing 0.5 μg/mL ethidium bromide at 80 V for 20 min. The bands were imaged using the gel documentation system.

#### In vitro studies

**Cell viability:** The effect of blank polymer and polyplex was determined using MTS (3-(4,5-dimethylthiazol-2-yl)-5-(3-carboxymethoxyphenyl)-2-(4-sulfophenyl)-2*H*-tetrazolium) assay (Cell Titer 96 Aqueous one solution, Promega, USA). A number of 4000 A549 cells per well was cultured in a 96-well plate at 37 °C in 5% CO_2_. Once the cells became confluent, the medium was removed and washed with phosphate buffered saline (PBS, pH 7.4) in order to remove the non-adherent cells. The polyplexes containing siRNA were dissolved in 100 µL of serum-free media and added to the cells such that the concentration of siRNA in each well was 100 nM. The medium was replaced with fresh medium after 4 h followed by incubation for specified periods of time (24 h or 48 h). MTS reagent (20 µL) along with 200 µL of serum-free media was added to each sample well and incubated at 37 °C for 2 h. The reaction was terminated with 10% sodium dodecyl sulfate (SDS) solution. The absorbance was read at 490 nm using a multimode reader (Epoch i2, Biotek, USA). For assessing the effect of VEGF silencing on the cytotoxicity of 5-FU, the cells were initially treated with the polyplex or with free siRNA at a siRNA concentration of 100 nM for 4 h. The medium was then replaced with fresh medium to which 400 μM of 5-FU was added and incubated for 48 h. The cell viability was then assessed using the MTS reagent as described above.

**Internalization studies:** Internalization of the polyplex in A549 cells was studied with Cy3 fluorophore-tagged siRNA. A549 cells at a seeding density of 10^5^ cells/well were cultured on a cover slip in a 6-well plate. When the cells reached confluency, medium was removed and non-adherent cells were removed by washing with PBS. The polyplexes containing fluorescent siRNA were added to 100 µL of serum-free media so that the final concentration of siRNA in the system was 100 nM. At pre-determined time points, the cells were stained with Hoechst 33258 and imaged with laser scanning confocal microscopy (Olympus FV1000, Tokyo, Japan).

**Gene expression analysis:** VEGF gene silencing was determined using reverse transcriptase-polymerase chain reaction (RT-PCR, AG22331, Eppendorf, Germany). About 3 × 10^5^ A549 cells were incubated with PVI, free siRNA and the polyplex. The siRNA concentration was maintained at 100 nM in all in vitro experiments. 48 h after incubation, the cells were harvested for RNA isolation. The time point was chosen based on earlier literature reports where the VEGF silencing efficiency was evaluated in different cell lines 48 h after treatment [4]. RNA was isolated using Trizol (Invitrogen, USA) and quantified using Nanodrop (Thermo Instruments, USA). The quantified RNA was then converted to cDNA using iScript cDNA synthesis kit (BioRad, USA). The cDNA was amplified using VEGF-specific primers and quantified using SYBR green (BioRad, USA). The relative gene expression was calculated using the ΔΔCt method. β-actin was used as the house-keeping gene. The sequences of the primers used in the study are given in Table 1.

**Table 1:** Primers used for gene expression studies.

primer	forward	reverse

VEGF	5′-TGCCCACTGAGGAGTCCAAC-3′	5′-TGGTTCCCGAAACGCTGAG-3′
β-actin	5′- CTCTTCCAGCCTTCCTTCCT-3′	5′-AGCACTGTGTTGGCGTACAG-3′

**Western blot analysis:** Total protein was isolated from the A549 cells using cell lysis buffer (1× RIPA buffer, PMSF, 1% protease and protease inhibitors cocktail, Cell Signaling Technology, USA) and quantified using Lowry’s method. An aliquot of the cell lysate containing 50 µg protein was loaded in 12% sodium dodecyl sulfate–polyacrylamide gel. The membrane was blocked for 1 h with blocking buffer (5% skimmed milk in Tris-buffered saline containing 0.1% Tween-20) followed by overnight incubation with primary antibody (VEGF antibody, dilution 1:500, Santa Cruz Biotechnology, USA) at 4 °C. The blots were then washed and incubated for 1 h with appropriate anti-mouse horseradish peroxidase-conjugated secondary antibodies (dilution 1:5000, Cell Signaling Technology, USA) at room temperature. The protein spots were visualized using tetramethyl benzidine/hydrogen peroxide (TMB/H_2_O_2_, Bio-Rad, USA) reagent. Membranes were stripped, reblocked, and re-incubated with the primary antibody against the housekeeping protein β-actin (Cell Signaling Technology, USA). Images were acquired using a gel documentation system (Fusion SoloX, Vilber Lourmat, France). The Bio-1D software was used for analysis of the images and the intensity of the bands was calculated. The background was normalized, and the intensity obtained for β-actin band was used to normalize the band intensity of the corresponding VEGF protein band.

**Flow cytometry:** Apoptotic cells were visualized using an Annexin V-FITC apoptosis detection kit (BD Biosciences, San Jose, USA) according to the manufacturer’s protocol. Briefly, cells were harvested 48 h after transfection, washed twice with PBS, and re-suspended in 300 μL of Annexin V binding buffer. Five microliters of FITC-conjugated Annexin V was added to the cell solutions, followed by adding 5 μL of propidium iodide (PI). After incubation for 15 min at room temperature in the dark, samples were immediately analyzed using a BD FACS Lyric – 4C flow cytometer (BD Biosciences, San Jose, USA). Data from approximately 1 × 10^5^ cells were analyzed by using the BD FACS suite software (BD Biosciences, San Jose, USA).

**Anti-angiogenesis assay:** HUVECs were seeded at a density of 2 × 10^5^/well in a 6-well plate. After 24 h, the medium was replaced with serum-free medium and left overnight. The change in morphology with formation of tubular network was monitored using an inverted microscope (Eclipse Ti, Nikon, Japan). Then, the cells were treated with the samples (free siRNA or polyplex) containing a final concentration of 100 nM siRNA and incubated for specified periods of time. The cells were imaged using an inverted microscope to observe the effect of the treatment on the tubular network.

#### Migration analysis

**Wound healing assay:** To evaluate the gene silencing effect of the polyplex, cell migration assays were performed using A549 cells. The cells were cultured in a 6-well plate with a seeding density of 10^5^ cells/well. After the cells attained confluency, the non-adherent cells were removed after removal of the medium and washed with PBS. The polyplex containing 100 nM anti-VEGF siRNA was added to 100 µL of serum-free media. After 4 h, the medium was replaced with fresh medium. A straight scratch was made in the well using a pipette tip to remove the cells in the scratch zone. Cell migration was observed after 48 h and the images were captured using light microscope. The migration rate was compared with untreated cells as well as cells treated with equivalent quantities of blank PVI nanoparticles and 100 nM of free siRNA. The relative migration ability of the cells in each case was calculated as follows:


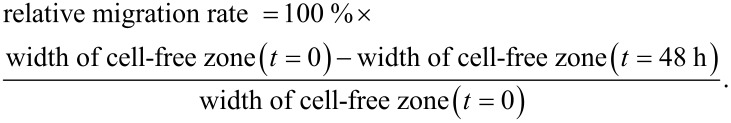


**Transwell assay using a Boyden chamber:** To quantify the migration, a Boyden chamber assay (HiMedia, USA) was performed using 8 μm pore sized transwells for A549 cells. After 48 h of treatment with free siRNA, polyplex or pristine polymer, the cells were trypsinized and re-suspended in serum-free media. For quantifying the migration, serum-free media with 40,000 cells were seeded in the upper compartment of the transwell chamber and the lower chamber contained complete media with 10% FBS. After 24 h, the cells migrated through the membrane were quick fixed with 100% cold methanol for 15 min at −20 °C and then rinsed with DPBS. The fixed cells were stained with 0.5% crystal violet for 20 min and washed with DPBS. The chambers were air-dried and observed using phase contrast microscopy and imaged. The numbers of cells were counted at ten different fields for quantification.

#### Microarray analysis

For performing microarray analysis, the quality of the total isolated RNA was checked using gel electrophoresis. The microarray experiment was performed using oligonucleotide microarrays (Genechip - Primeview human, Affymetrix, USA) that can detect the expression of 49,372 genes. Typically, 500 ng of RNA was converted to double-stranded cDNA. The biotin-labeled complementary RNA (cRNA) was amplified using in vitro transcription (IVT) of the double-stranded cDNA template using T7 RNA polymerase. 12 μg of the purified cRNA was fragmented using divalent cations at elevated temperature. The labeled fragmented sample was loaded into Primeview® Genechip and the chip was hybridized overnight at 45 °C, 60 rpm in the Genechip Hybridization Oven 645. The chip was washed and stained in the Fluidic Station FS450. The chip was then scanned using a Genechip Scanner 3000 7G. The data analysis to identify differentially regulated genes in the cells treated with polyplex when compared with cells treated with free siRNA was performed using Transcriptome Analysis Console v3.0 (Affymetrix, USA.) Fold values above 2.0 were considered as up-regulated and fold values below −2.0 were considered down-regulated.

#### Statistical Analysis

The statistical significance of the data obtained for the cell viability was assessed by one-way ANOVA, gene expression, protein expression and migration assay. The level of significance was found using Kruskal–Wallis statistical test or Bonferroni comparison test; *p* < 0.05 was considered significant.

## Results and Discussion

### Complexation of siRNA

The content of free siRNA progressively decreases with increasing polymer concentration while the complexed form exhibits a corresponding increase in intensity. The maximum intensity was observed at a 4:1 ratio of polymer to siRNA. A slight amount of uncomplexed RNA is observed even at the polymer/siRNA ratio of 4:1, which corresponds to a N/P ratio of 250. These results were confirmed by the ribogreen assay where it was observed that about 66% of siRNA was complexed at a 4:1 ratio of PVI/siRNA. The imidazole nitrogen atoms are protonated at acidic pH values [22] while the complexation was carried out at a physiological pH value of 7.4, at which considerably less imidazole nitrogen atoms are protonated. This leads to high N/P ratios required for complexation. Although the polyplex ratio of 8:1 also exhibits superior complexation, the 4:1 ratio was preferred as very high concentrations of the polymer may impede the release of siRNA.

**Figure 1:**
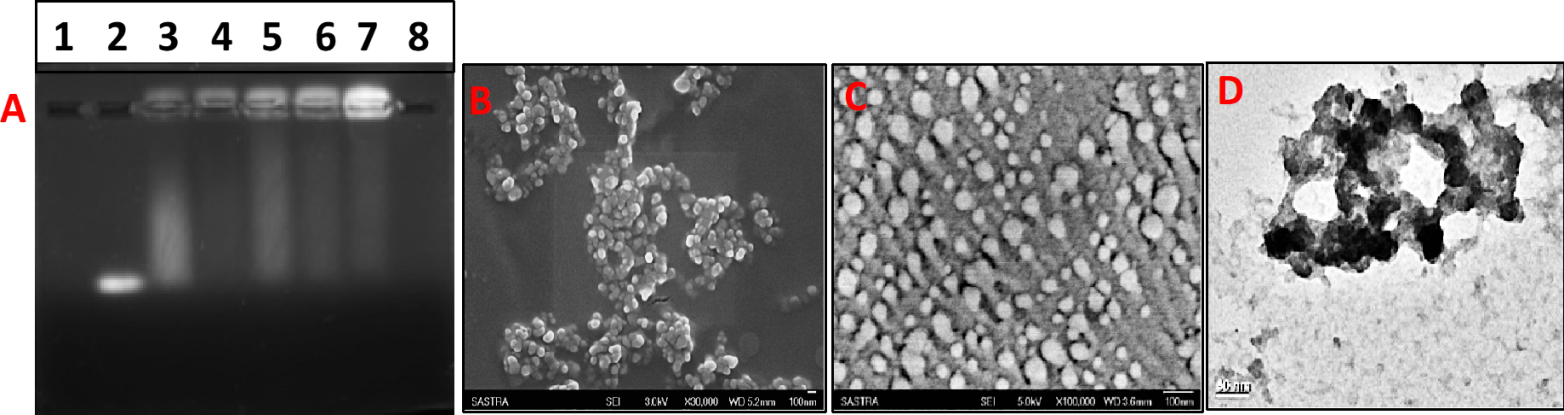
(A) Gel retardation assay for polyplexes formed with different ratios (v/v) of PVI/siRNA. Ethidium bromide dye (2 µg/mL) was used. The bands were visualized using UV transillumination. Lane 1: blank; lane 2: free siRNA; lane 3: polyplex formed with a 1:1 ratio of PVI/siRNA; lane 4: polyplex formed with a 2:1 ratio of PVI/siRNA; lane 5: polyplex formed with a 3:1 ratio of PVI/siRNA; lane 6: polyplex formed with a 4:1 ratio of PVI/siRNA; lane 7: polyplex formed with a 8:1 ratio of PVI/siRNA; lane 8: blank. (B) Scanning electron micrograph of blank polymer nanoparticles. (C) Scanning electron micrograph of the polyplex formed with a polymer/siRNA ratio (v/v) of 4:1. (D) Transmission electron micrograph of the polyplex formed with a polymer/siRNA ratio (v/v) of 4:1.

The complexation of siRNA with PVI was determined at different polymer/siRNA ratios and the results are presented in Figure 1A.

Figure 1B–D shows scanning electron micrographs and a transmission electron micrograph of the polyplex formed at a PVI/siRNA ratio of 4:1. Formation of spherical nanoparticles in the size range of 80 to 120 nm is clearly discernible from the electron micrographs. The hydrodynamic diameter of the blank PVI nanoparticles measured using dynamic light scattering was found to be about 237 ± 34.6 nm. The zeta potential measured for the blank PVI nanoparticles in HEPES buffer at pH 7.4 was found to be 16.2 ± 2.76 mV while it reduced to 12.3 ± 0.92 mV after complexation with siRNA. The shift in the zeta potential values indicate the formation of the PVI–siRNA polyplex, which has resulted in an alteration in the surface charges.

**Figure 2:**
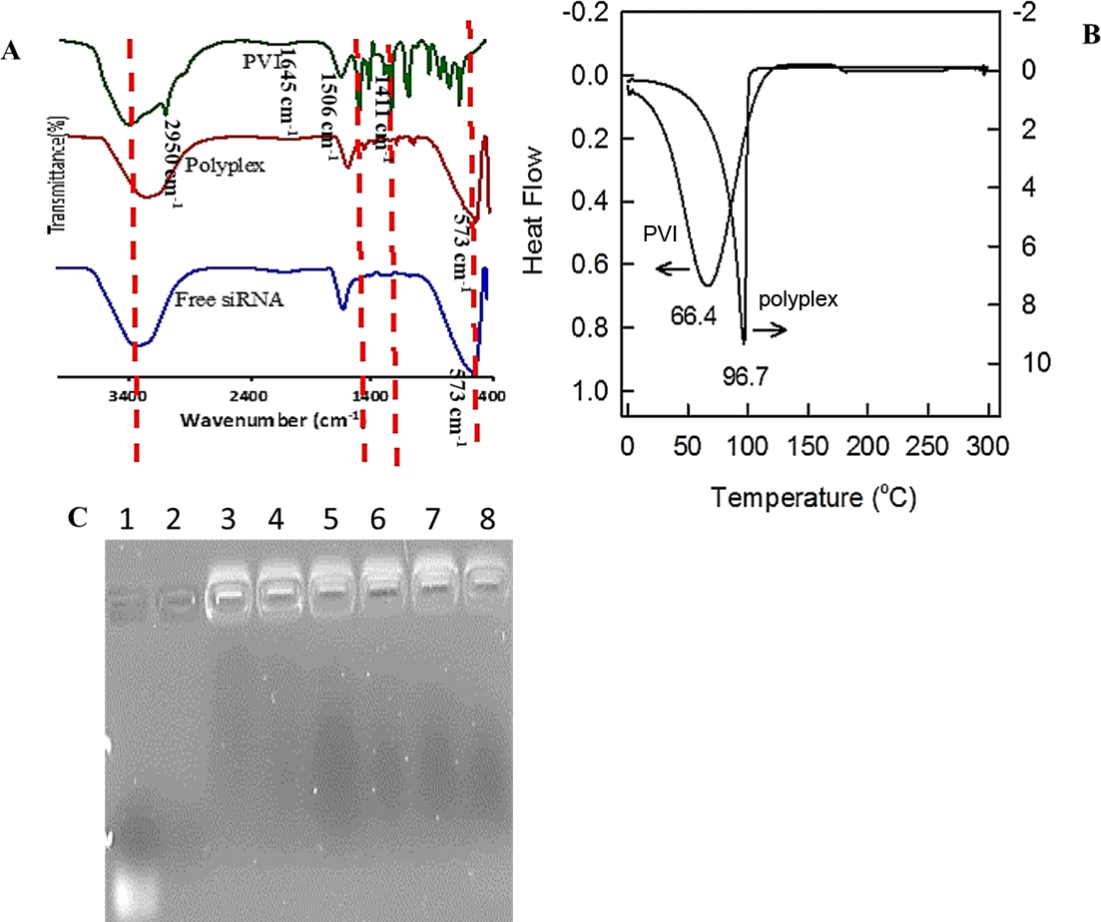
(A) FTIR spectra of free siRNA, PVI and the polyplex recorded in ATR mode between 4000 and 400 cm^−1^. (B) Heat flow profiles of pristine PVI and polyplex in nitrogen atmosphere at a scan rate of 5 °C·min^−1^. (C) Heparin displacement assay for the polyplex at different concentrations of heparin. Lane 1: free siRNA; lane 2: 60 ng/mL heparin only; lane 3: heparin/polyplex ratio (w/w) of 0.5; lane 4: heparin/polyplex ratio (w/w) of 1, lane 5: heparin/polyplex ratio (w/w) of 1.5; lane 6: heparin/polyplex ratio (w/w) of 2; lane 7: heparin/polyplex ratio (w/w) of 2.5; lane 8: heparin/polyplex ratio (w/w) of 3. The absence of free siRNA in the polyplex lanes treated with heparin shows the stability of the complex formed.

The complexation of siRNA with PVI was investigated by using FTIR and DSC (Figure 2). The FTIR of PVI shows vibration bands at 2950 cm^−1^ (imidazole C–H stretching vibrations) and at 1645, 1506 and 1411 cm^−1^ (imidazole C–N stretching vibrations). The N–H in-plane bending vibrations are observed at 1235 cm^−1^. The polyplex also shows stretching vibrations at 1635, 1501 and 1427 cm^−1^ (C–N vibrations), and at 1235 cm^−1^ (N–H in-plane bending vibrations). The band appearing at 573 cm^−1^ in the FTIR spectra of the polyplex and blank siRNA may be attributed to the phosphate groups of the oligonucleotide clearly confirming the complexation.

The differential thermal calorimetric profile (Figure 2B) reveals that the melting point of the pristine polymer is about 65 °C. Upon complexation with siRNA the melting point shifts to 98 °C. This can be attributed to the electrostatic interactions between the anionic phosphate moieties in the siRNA with the cationic imidazole groups in the polymer resulting an increase in the melting point of the polyplex.

The stability of the polyplex in serum is a major factor influencing its therapeutic efficacy. The serum proteins could dissociate the siRNA–polymer complex reducing the delivery efficiency. Heparin is a glycosaminoglycan associated with the inhibition of the coagulation process by activating anti-thrombin [23]. The sulfate groups present in heparin compete with the oligonucleotide sequences to associate with the polymer chains and based on the relative strength of the electrostatic interactions between the oligonucleotide and polymer, heparin may succeed in displacing the oligonucleotide from the polyplex. Our results show an absence of free siRNA after successive addition of heparin to the polyplex. The stability of the polyplex is maintained even when the heparin/polyplex ratio (w/w) reaches 3. This indicates good serum stability of the polyplex.

### In vitro studies

**Figure 3:**
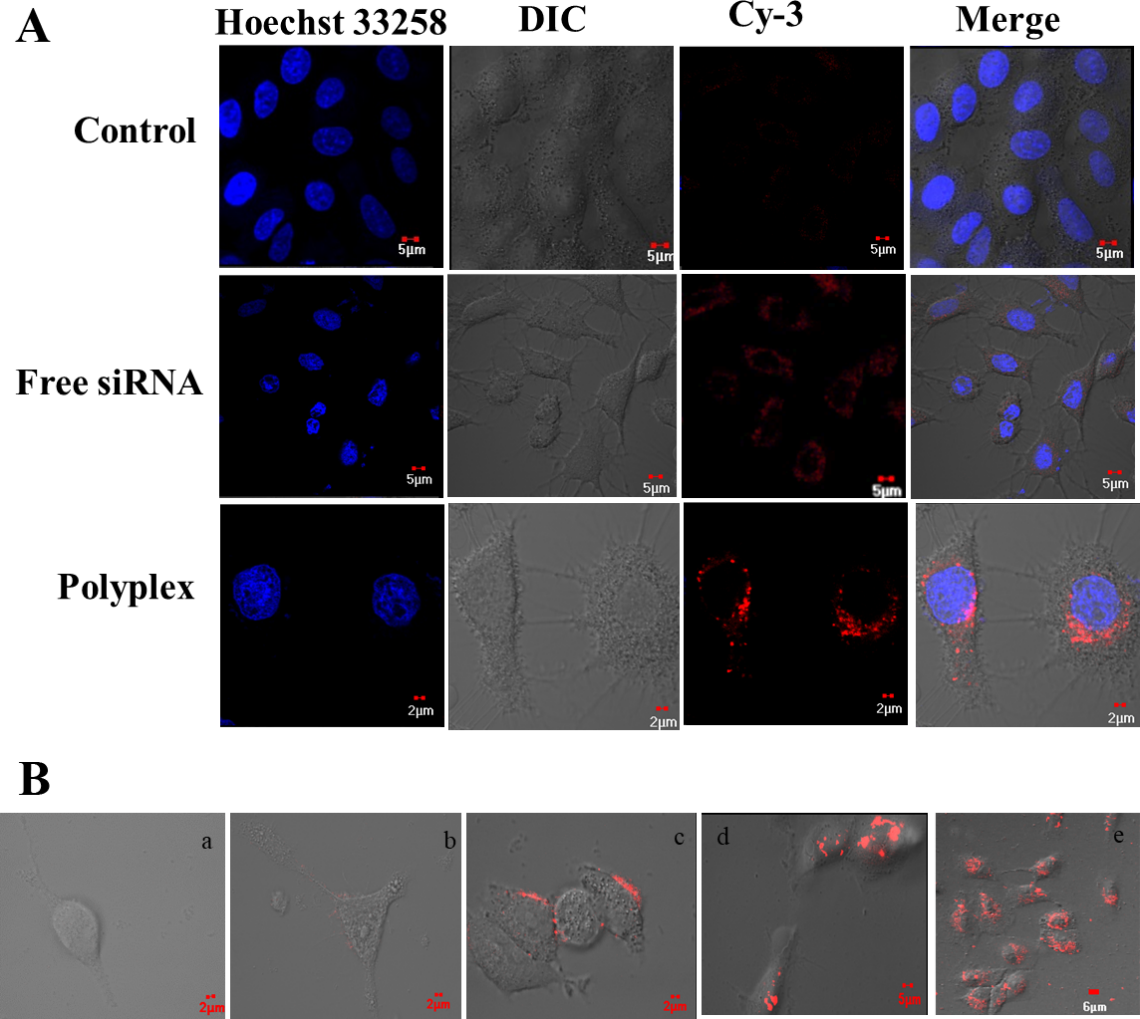
(A) Intracellular uptake of the polyplex monitored by using laser scanning confocal microscopy after 4 h of treatment with Cy3-labeled anti-VEGF siRNA (emission wavelength = 568 nm) at a final siRNA concentration of 100 nM and complexation at a volume ratio of 4:1. Untreated cells were used as control. Nuclei were stained with Hoechst 33258 (blue, emission wavelength = 405 nm). (B) Intracellular uptake of the polyplex after different periods of time, a: control, b: after 30 min, c: after 1 h, d: after 4 h, e: after 16 h.

The internalization of the polyplex in A549 cells was determined and the results are presented in Figure 3. It is observed that the PVI polyplexes accumulate on the membrane surface after 15 min and are starting to be internalized into the cells after 30 min. 2 h after administration, the polyplexes are found inside the cells and remain there even after 16 h (Figure 3B). The polyplexes were found in the cytosol, which is preferable for gene silencing as the RNA-induced silencing complex (RISC) [24] is formed in the cytoplasm.

**Figure 4:**
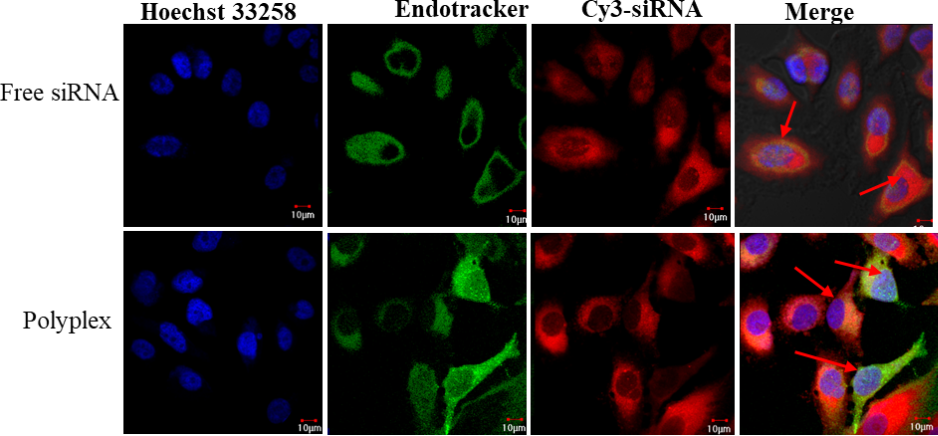
The endosomal escape of the polyplex after 4 h of incubation in A549 cells visualized using endotracker (stains early endosomes green) and the red fluorescence from Cy-3 labeled siRNA observed with confocal laser scanning microscopy. The co-localization of the Cy3-labeled siRNA and endosome is observed as yellow fluorescence (red arrows). The green emission was recorded at 488 nm while the red emission was captured at 568 nm. The blue fluorescence of Hoechst 33258 was recorded at 405 nm.

Most gene delivery systems become ineffective in delivering their cargo due to their inability to escape the endosome in cells. To investigate ability of the PVI polyplexes to escape from endosomes, their co-localization with the endosomes was investigated and the results are presented in Figure 4.

The green fluorescence of the endosomes and the red fluorescence of the fluorophore-tagged siRNA are perfectly merged indicating that the free siRNA is unable to escape from the endosome. This is expected as it has been established earlier that double-stranded oligonucleotides tend to accumulate in the endosomal compartment and very few manage to reach the cytosol [25]. The polyplex-treated cells reveal several zones of red fluorescence localized at regions distinct from the green fluorescence. This suggests that a fraction of the polyplex has escaped from the endosomal compartment, which is advantageous for gene delivery applications. The imidazole moieties in PVI can neutralize the acidic pH value, which in turn can aid their endosomal escape through the proton sponge mechanism, through altering the membrane permeability or through by-passing the endosomes [26]. However, the confocal images also reveal that a fraction of the polyplex identified by the red fluorescence also co-localizes with the endosomes with green fluorescence. This suggests that a part of the polyplex remains in the endosome and may not be able to contribute to the silencing.

### Gene expression analysis

**Figure 5:**
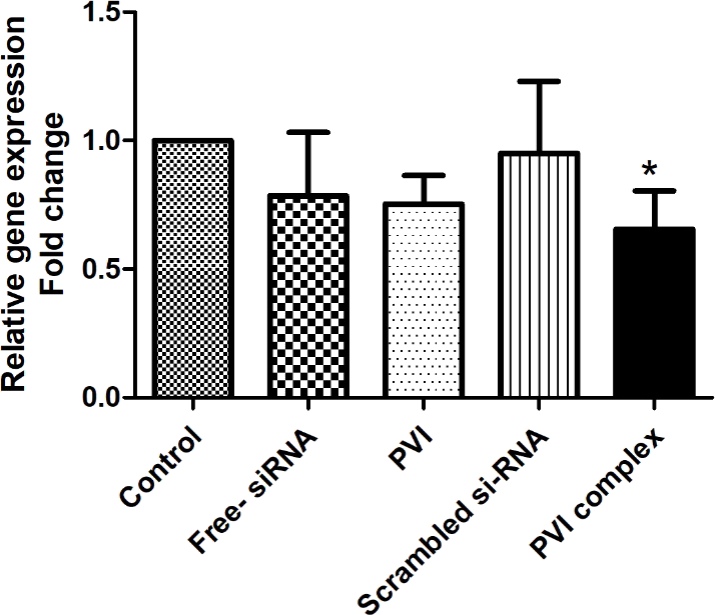
Expression of vascular endothelial growth factor (VEGF) mRNA in A549 cells analyzed by RT-PCR. The fold change was calculated using the ΔΔCt method. The results are represented as mean ± SD and analyzed using one-way ANOVA followed by post-hoc Bonferroni comparison test (*n* = 3, * *p* < 0.05) vs control.

To evaluate the efficiency of the polyplex to silence VEGF in lung cancer cells, VEGF gene expression analysis using RT-PCR was performed. It was found that the polyplex-treated cells showed a significant reduction in the expression levels of VEGF 48 h after treatment. The cells treated with free siRNA did not show a significant reduction in the VEGF expression levels (Figure 5). This difference in silencing efficiency may be attributed to the better ability of the polyplex to internalize into the cells and a higher fraction of the polyplex being able to escape the endosome. Scrambled siRNA did not show any significant change in the VEGF expression and was comparable to the control.

### Western blot studies

**Figure 6:**
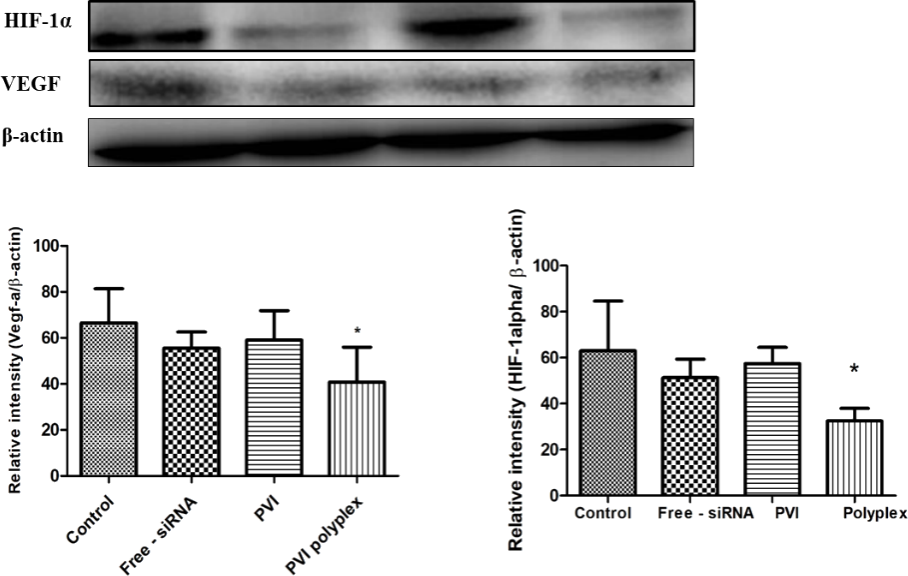
VEGF, HIF-1alpha and β-actin protein expression levels, obtained from Western blot, in A549 cells after different treatments. The expression of VEGF and HIF-1 alpha was normalized to the corresponding expression of β-actin, which was used as house-keeping gene. Data shown as mean ± SD of triplicate independent experiments; *p < 0.05 compared to control.

Figure 6 shows the VEGF protein levels quantified through Western blot analysis from cells treated with free siRNA, blank PVI, and polyplex. A significant decrease in the VEGF levels was observed in cells treated with the polyplex suggesting that the polyplex leads to a better internalization of the siRNA. This is also reflected in the differential modulation of gene targets associated with VEGF in the microarray analysis. The superior silencing of VEGF by the polyplex is also reflected in the reduced levels of hypoxia-inducible factor-1 alpha (HIF-1α). HIF-1α is expressed under oxygen-deficient conditions and has been found to induce the expression of VEGF thereby promoting angiogenesis [27]. Recently, it has been found that HIF-1α and VEGF levels regulate each other through a competing endogenous RNA pathway involving miRNA [28]. Our data correlate with this finding as we find that silencing VEGF also leads to a decrease in the HIF-1α levels.

**Figure 7:**
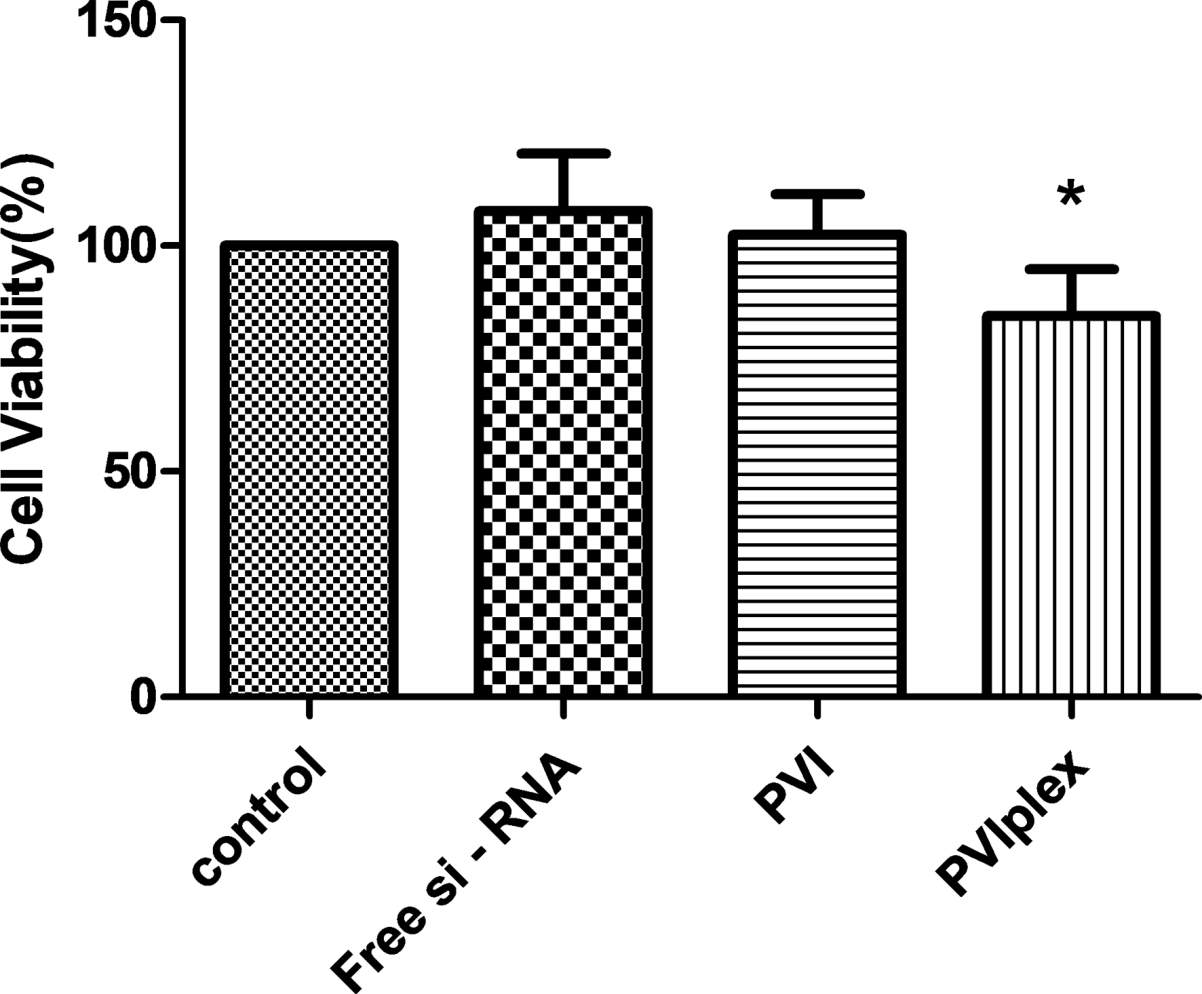
Cell viability of A549 cancer cells after treatment with carrier systems (blank carrier and the polyplex obtained after complexation with anti-VEGF siRNA). The A549 cells displayed a lower viability after 48 h of treatment (*p* < 0.05 vs control, *n* = 5).

The effect of the polyplex on the viability of A549 lung cancer cells was investigated after 48 h and the results are shown in Figure 7. After 48 h, the cells treated with 100 nM of free siRNA and blank polymer exhibited viabilities exceeding that of the untreated control cells while those cells treated with the polyplex exhibited a decreased viability.

VEGF inhibition has been earlier reported to decrease the proliferation of cancer cells due to its ability to interfere with the MAPK and PI3K/Akt signaling pathways [29], which could be reflected in the viability values. The absence of any inhibition in the cell viability by the blank polymer indicates its cyto-compatibility while the lack of activity in the case of free siRNA may be due to its inability to escape from the endosome. The complexation of the siRNA with PVI results in better internalization and it promotes endosomal escape thereby aiding to form a RISC to cleave VEGF mRNA.

### Flow cytometry analysis

**Figure 8:**
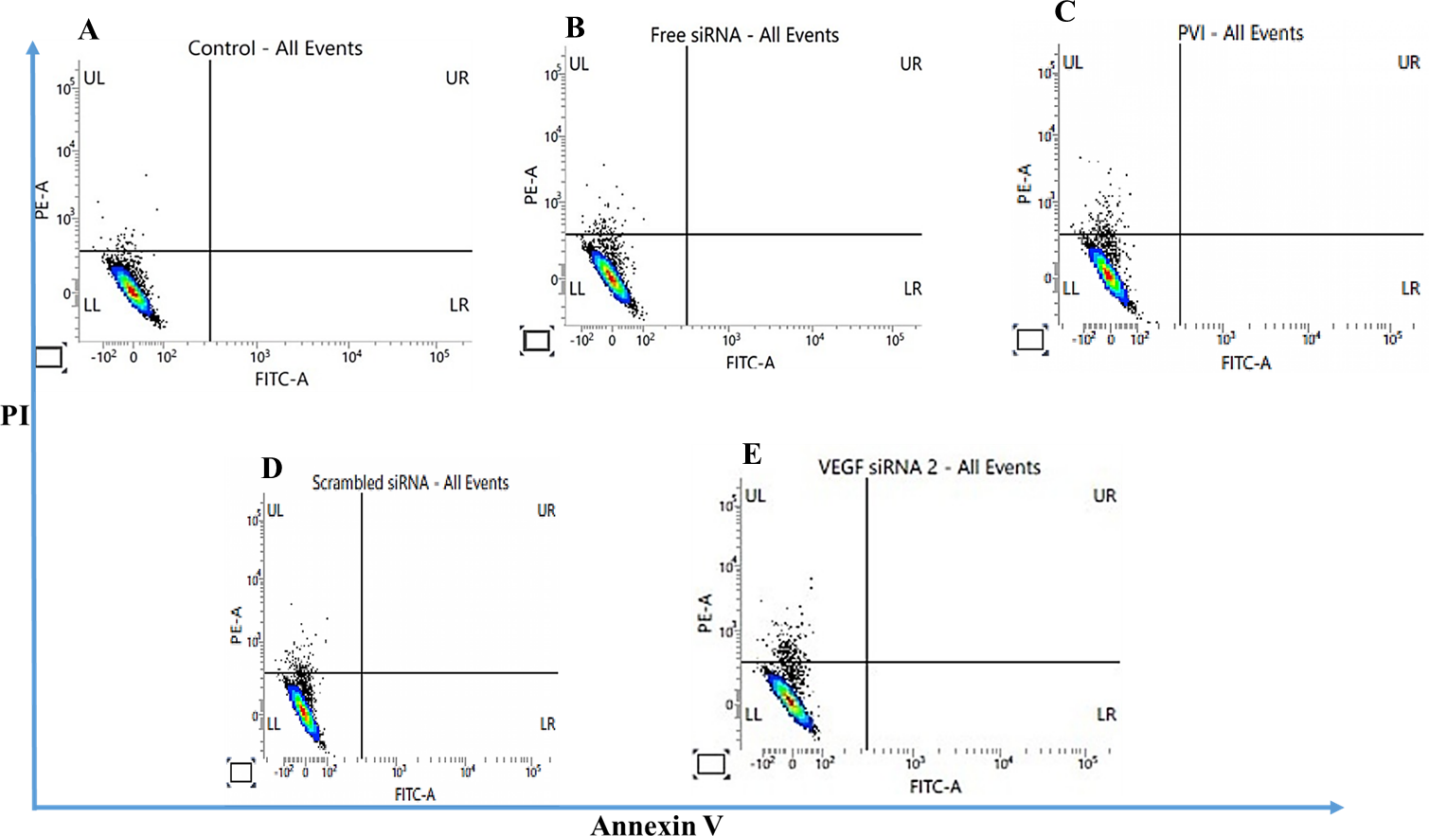
Flow cytometry analysis of A549 cells treated for 4 h with VEGF siRNA and analyzed 48 h after treatment. The results were compared with control and cells treated with scrambled siRNA. (A) Control; (B) free siRNA; (C) PVI; (D) scrambled siRNA; (E) VEGF siRNA. Cells were stained using FITC-conjugated Annexin V and propidium iodide (PI).

The number of apoptotic cells after treatment with VEGF siRNA was determined by using flow cytometry. The cell viability results revealed that after 48 h of treatment with VEGF siRNA, A549 cells treated with the polyplex exhibited a reduction in the viability when compared to control and scrambled siRNA. A similar trend was observed in the flow cytometry data where the number of apoptotic cells was found to be higher after treatment with the PVI polyplex than after treatment with free siRNA or scrambled siRNA (Figure 8).

**Figure 9:**
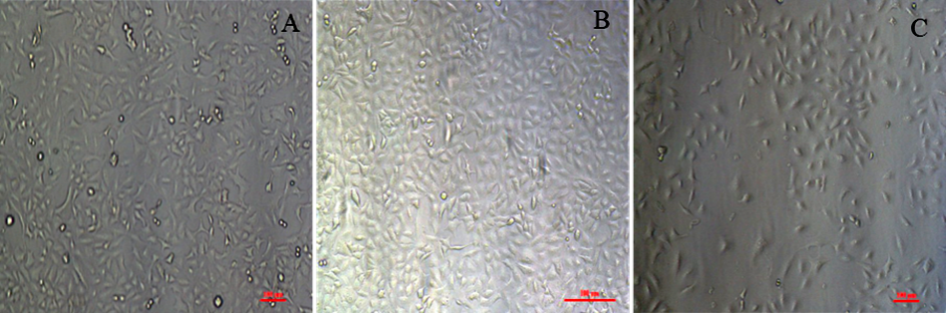
Morphology of A549 cells after treatment with blank PVI nanoparticles or polyplex. (A) Control; (B) blank polymer nanoparticles; (C) polyplex. The scale bars represent 100 μm.

The morphology of the cells treated with the blank polymer and the polyplex are compared with the untreated control cells is presented in Figure 9. Although there are no significant changes visible in the morphology after treatment with siRNA complexes, a subtle reduction in the tendency of the cells to interact with each other is discernible in the cells treated with the PVI–siRNA complex where the cells appear to lose contact with each other. This may be suitable for cancer therapy as the treatment may retard spheroid and stroma formation. VEGF signaling has been connected to an activation of focal adhesion kinases and paxillin leading to cell morphology changes promoting migration [30]. Therefore, the inhibition of VEGF leads to a reduction in the formation of cell–cell junctions that are a primer for cell migration. The treatment with blank polymer did not show any changes in the clustering of cancer cells indicating that the observed change is solely due to VEGF inhibition through the PVI polyplex.

### Cell migration measurements

**Figure 10:**
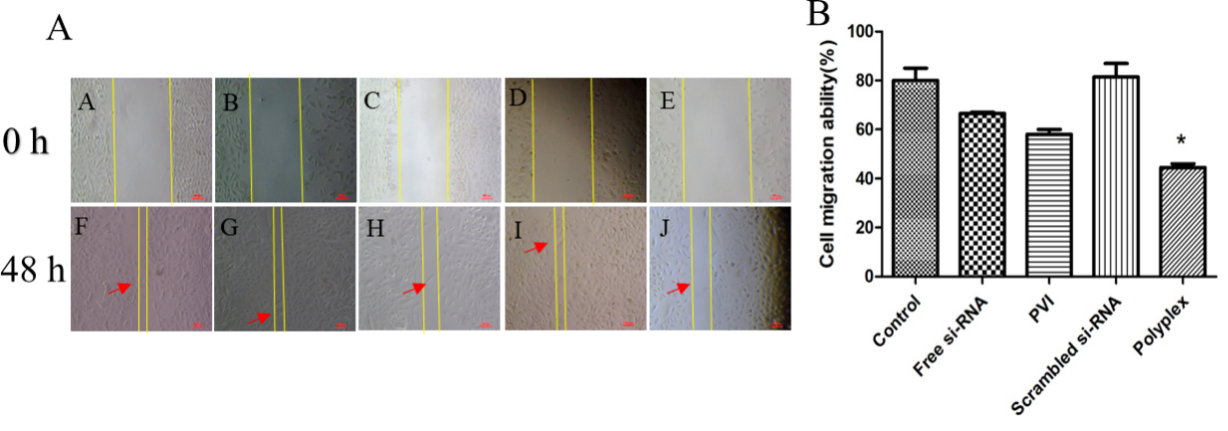
(A) Migration of A549 cells 48 h after treatment with free siRNA, blank polymer nanoparticles, polyplex with scrambled siRNA, and polyplex with VEGF siRNA. VEGF silencing slows down the migration of A549 cells. Cells were exposed to the anti-VEGF siRNA for 4 h. (B) Migration rate of the A549 cells 48 h after treatment, *n* = 3, *p < 0.05 vs control.

**Figure 11:**
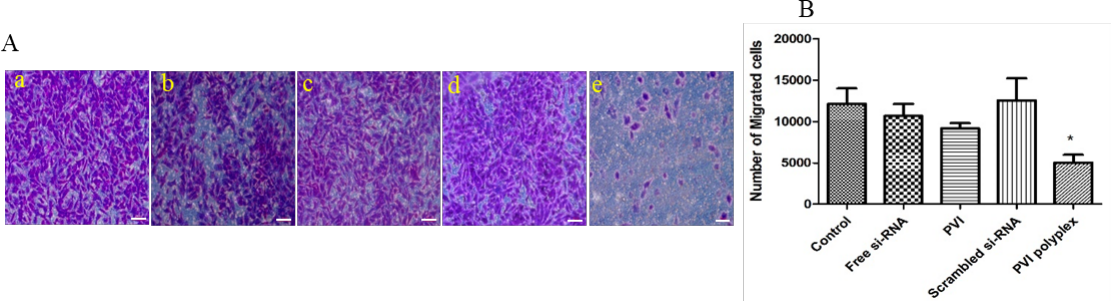
(A) Migration of A549 cells analyzed using Boyden chamber assay 48 h after treatment. a: Control; b: free siRNA; c: blank polymer nanoparticles; d: polyplex with scrambled siRNA; e: polyplex with VEGF siRNA. VEGF silencing slows down the migration of A549 cells. The cells were stained using 0.5% crystal violet for visualization and images were taken using phase-contrast microscopy. The scale bars represent 50 μm. (B) Migration of the A549 cells 48 h after treatment, *p < 0.05 vs control.

The effect of VEGF siRNA silencing on cell migration was investigated using cells treated with free siRNA or polyplex for 48 h (Figure 10). It is observed that the scratched gap was filled more slowly in cells treated with free siRNA when compared with the control cells. This was even more delayed in cells treated with the polyplex and pristine PVI. It is likely that the polymer also exhibits a retarding effect that in combination with VEGF silencing interferes with the migration potential of the cancer cells. The cells treated with scrambled siRNA sequence did not show any significant difference from the control (untreated) cells in the migration ability clearly indicating that silencing VEGF influences cell migration. Similar results have been reported for VEGF silencing in other cancer cells including hepatocellular carcinoma and pancreatic cancer [31]. Also, Boyden chamber assay measurements were carried out (Figure 11) in which the same trends were observed.

### Cell viability measurements

**Figure 12:**
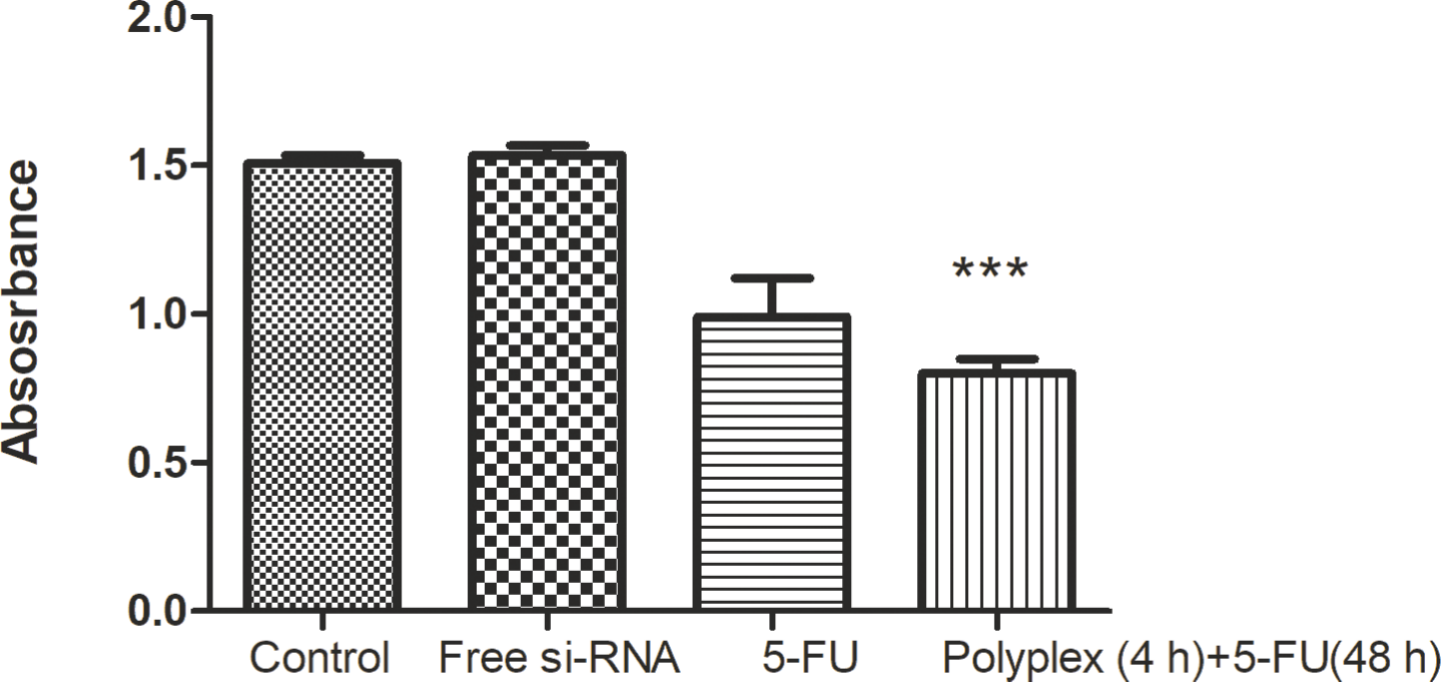
Viability of A549 cells after silencing VEGF using free siRNA or the polyplex for 4 h followed by treatment with 5-FU for 48 h. A final concentration of 100 nM siRNA and 400 µM of 5-FU were used in A549 cells and shown as mean ± SD (*n* = 3). ***p < 0.05 compared with the control.

VEGF inhibition can also be used in combination with chemotherapeutic agents to enhance their therapeutic efficacy. The ability of the polyplex to alter the cell viability of A549 cells treated with 5-FU was investigated and the results are represented in Figure 12. The cells were first treated with the polyplex for 4 h after which the medium was replaced. Different concentrations of 5-FU were added, and the viability was determined after 48 h. Imidazole and its derivatives have been earlier shown to inhibit the migration and the invasiveness of cancer cells [32]. Our results show that also PVI with its multiple imidazole groups can retard the migration of A549 cancer cells.

It is observed that the cell viability of A549 lung cancer cells decreased when pre-treated with the polyplex for 4 h followed by treatment with 5-FU for 48 h. Free 5-FU led to a decrease in cell viability by 77% at a concentration of 400 μM while free siRNA reduced the viability to 80% at a concentration of 100 nM. In the presence of VEGF-loaded polyplex, the same concentration of 5-FU reduced the viability of A549 cells to 57% indicating that VEGF silencing sensitizes the cells to 5-FU. The use of PVI-based polyplexes with VEGF siRNA have not been investigated for their chemosensitizing properties. However, similar observations have been made in hepatoma cells that were sensitized to doxorubicin following VEGF silencing [33].

**Figure 13:**
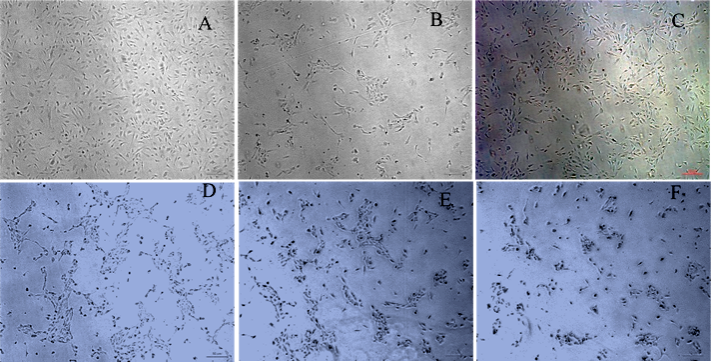
Morphology of HUVECs cultured in serum-free medium after (A) 0 h, (B) 24 h, and (C) 48 h; (D) control (E, F) HUVECs after 4 h of treatment with PVI–siRNA complex (100 nM).

VEGF is a key factor that initiates angiogenesis and hence its silencing is expected to influence the formation of new blood vessels. HUVECs have been extensively used to monitor the effects of pro-angiogenic and anti-angiogenic factors. HUVECs respond to serum starvation by enhancing the expression of HIF-1α, which activates VEGF. This is manifested by distinct changes in the morphology of the HUVECs, which become elongated and associate to form chains and tubular structures. Figure 13 shows the changes that occur in the morphology of HUVECs after 0, 24 and 48 h in serum-free medium. The high-magnification image of HUVECs after 48 h shows the formation of elongated tubular assemblies that are characteristic of neo-angiogenesis. Figure 13 shows the effect of PVI polyplex treatment on the morphology of HUVEC cells in serum-free medium. A distinct alteration in the morphology of the HUVECs is observed characterized by the absence of distinct elongation and tube formation in the cells clearly indicating the effect of VEGF inhibition.

Earlier studies have shown that VEGF binds to α_9_β_1_ integrin on the cell surface, which mediates the formation and migration of endothelial cells through Src and focal adhesion kinase [34]. Therefore, the silencing of VEGF retards tube formation and extension of the endothelial cell processes as observed in the case of the polyplex-treated HUVECs in the present study. The anti-angiogenesis effect of VEGF silencing has been well documented in literature [35]. Our studies concur with these reports and show that the VEGF siRNA delivery through a PVI polyplex is effective in inhibiting tube formation in endothelial cells.

### Microarray analysis

**Figure 14:**
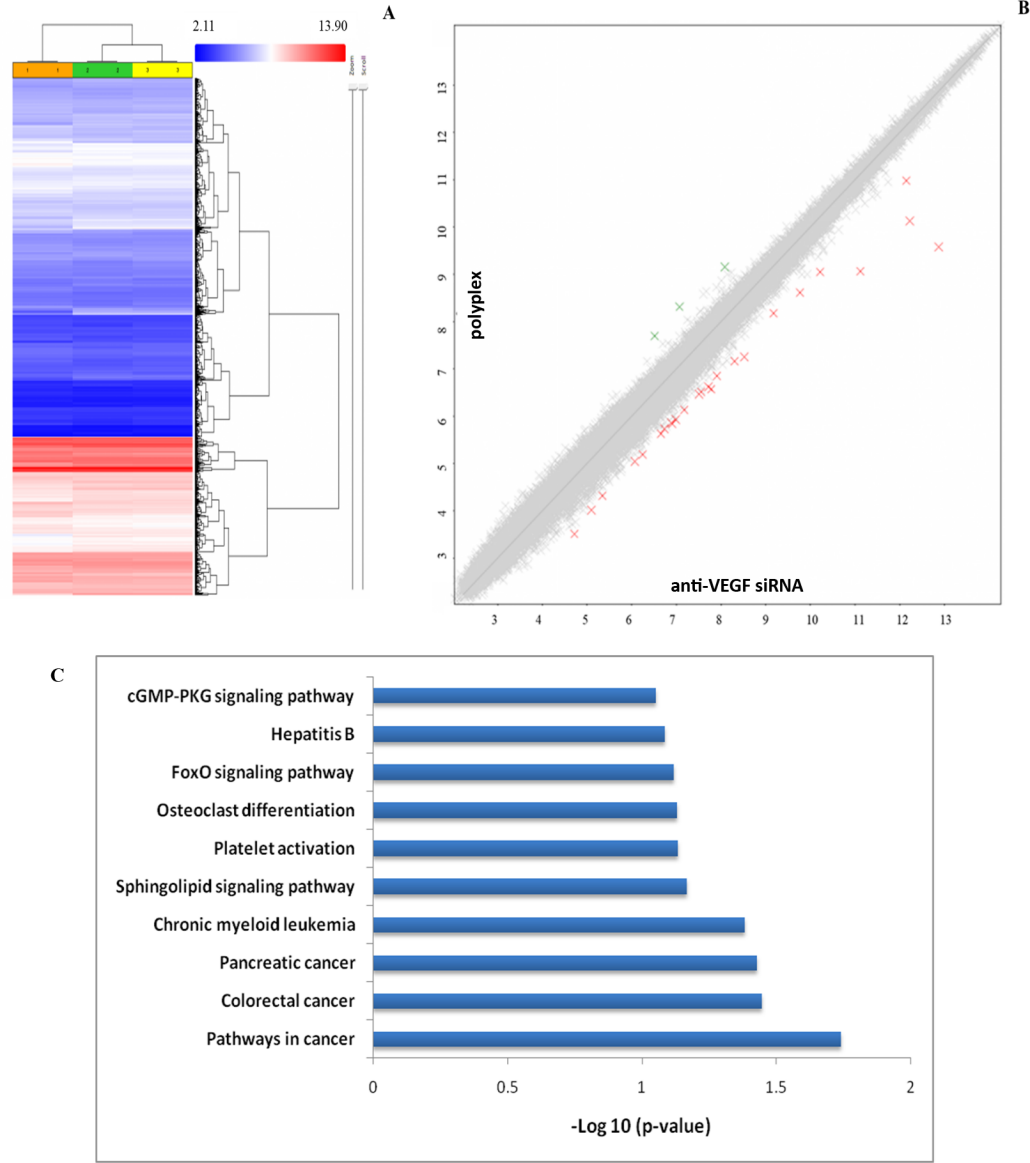
(A): Hierarchical clustering obtained from the microarray analysis (49,372 genes). Each row represents various genes and each column represents different samples. Column 1: (orange): control; column 2 (green): free siRNA, and column 3 (yellow): polyplex-treated cells. All experiments were carried out in replicates. (B) Scatter plot representing the gene expression profile of the polyplex as a function of that of anti-VEGF si-RNA (2) obtained from the microarray analysis. Total number of genes expressed: 49,372; green crosses: up-regulated genes; red crosses: down-regulated genes; grey crosses: genes with unchanged expression levels. (C) The KEGG pathway indicating the number of genes modulated by the polyplex treatment in A549 cells when compared with to the treatment with free siRNA.

VEGF has been implicated in a variety of signaling pathways in cancer that are now being revealed through gene and protein expression studies. To identify the effect of VEGF silencing on the various gene targets in A549 cells, a microarray analysis was performed and the relative change in expression levels of genes in cells treated with free siRNA and the polyplex was compared. Figure 14 shows the microarray data obtained for the genes deregulated by treatment with the polyplex in A549 cells after 48 h compared to cells treated the free siRNA and untreated cells. A fold change of 2 or above was considered for analysis.

Table 2 lists the gene targets that were altered by the polyplex treatment when compared to the cells treated with free siRNA. It is observed that TFF1 was found to be up-regulated. The role of TFF1 in cancer remains controversial but many reports have demonstrated that TFF1 serves as a tumor suppressor gene that inhibits cancer cell proliferation and migration in epithelial cancers such as gastric, breast and pancreatic cancer [36]. Recent experimental evidence has revealed that TFF1 when complexed with TFIZ1 exhibits tumor-suppressing activity while it transforms into a tumorigenic molecule when present in the uncomplexed state [37]. Among the genes that are down-regulated is PHF6 (PHD finger protein 6). This gene is differently expressed in different types of cancer. It was found that this gene is overexpressed in several epithelial cancers including breast and colorectal and serves as an oncogene [12]. The down-regulation may therefore be a positive indicator for lung cancer therapy. The silencing of PHF6 has been shown to inhibit the migration of hepatocellular cancer cells [38]. Other gene targets that are down-regulated are TGFBR1, the transforming growth factor beta receptor 1 and Akt3 [39–40]. TGF-β is involved in the proliferation of cancer by activating the phosphorylation of SMAD and subsequent nuclear translocation of transcription factors [39]. Interestingly, TGF-β has been implicated in angiogenesis through the activation of VEGF. TGF-β has also been implicated in promoting cancer cell migration [41]. Our migration assay data shows that the polyplex retards migration, which suggests that VEGF-mediated silencing by the polyplex and down-regulation of PHF3 and TGF-β could have contributed to this retardation in migration of the A549 cells. Akt has three isoforms that have been identified to possess different effects on the progression and invasiveness of lung cancer [42]. A recent report has demonstrated a direct relationship between the expression levels of Akt3 and VEGF [43]. Therefore, the reduced Akt3 expression in cells treated with the polyplex is a direct consequence of VEGF silencing. A related target that is suppressed in the polyplex-treated cells is GREM1 gene that encodes for Gremlin1, a key protein in the TGF-β signaling pathway, which is overexpressed in many types of cancer including lung cancer [44]. Gremlin1 is involved in the survival of the tumor cells and promotes the formation of the stromal barrier [44]. It has also been identified as an agonist of VEGF and its receptor VEGFR2 [44]. Therefore, the VEGF silencing of the polyplex is manifested through GREM1 down-regulation.

**Table 2:** Genes that were regulated by the polyplex.

no.	fold change	gene	description

1	−2.08	GREM1	Gremlin 1, DAN family BMP antagonist
2	−2.09	GNA13	guanine nucleotide binding protein (G protein), alpha 13
3	−2.01	PHF6	PHD finger protein 6
4	2.35	TFF1	trefoil factor 1
5	−2.04	BROX	BRO1 domain and CAAX motif containing
6	2.09	ZNF440	zinc finger protein 440
7	2.28	CEMIP	cell migration inducing protein, hyaluronan binding
8	−2.06	AKT3	v-Akt murine thymoma viral oncogene homolog 3
9	−2.04	TGFBR1	transforming growth factor, beta receptor 1
10	−2.32	TOR1AIP2	torsin A interacting protein 2
11	−2.06	FAM169A	family with sequence similarity 169, member A
12	−2.13	UBAP2	ubiquitin associated protein 2
13	−2.08	LCLAT1	lysocardiolipin acyltransferase 1
14	−2.14	CHP1	calcineurin-like EF-hand protein 1
15	−2.06	RFC5	replication factor C (activator 1) 5, 36.5kDa

The down-regulation of CHP-1 (calcineurin b homologous protein 1) gene in cells treated with the polyplex is also linked to the inhibition of angiogenesis. Indirect evidence from earlier reports implicates that the inhibition of CHP-1 is linked with the inhibition of HIF-1α, a key target in the angiogenesis pathway involving VEGF. The polyplex-treated cells had decreased levels of GNA13, UBAP2, RFC5, LCLAT1 and BROX genes. GNA13 is a target that has been associated with the proliferation and metastasis of many types of cancers [45]. Recent evidence has shown that high levels of GNA13 expression mediate angiogenesis through the elevation of VEGF levels. Our data reveal that the inhibition of VEGF can also suppress the GNA13 expression, which in turn could be beneficial for controlling the proliferation and invasion of lung cancer. LCLAT1 (lysocardiolipin acyltransferase1) has been implicated in the regulation of cardiolipin, a key membrane phospholipid. LCLAT1 has been associated with establishing endothelial lineages [46]. Independent studies have revealed elevated levels of LCLAT1 in colorectal and lung cancers that augments the unregulated proliferation and metastasis of the cancer cells [46]. Though direct involvement of LCLAT1 in modulating VEGF levels is yet to be established, studies have revealed a connection between mitochondrial dysfunction and VEGF expression [47]. LCLAT1 has been independently shown to cause mitochondrial dysfunction mediated through oxidative stress. Hence, it is likely that the down-regulation of VEGF may have contributed to the reduced expression of LCLAT1 in the polyplex-treated cells. RFC5 (replication factor C 5) is generally associated with the proliferation of cell nuclear antigen (PCNA) [48] and has also been implicated in DNA damage repair [48]. A recent study has identified RFC5 as a novel oncogene in lung cancer [49]. A Direct connection between RFC5 levels and VEGF expression is yet to be obtained. Nevertheless, independent studies have shown that RFC5 is regulated by FOXM1 (Forkhead box M1) while FOXO3 serves as an antagonist to FOXM1 and VEGF in lung cancer [50]. Based on this evidence, it may be inferred that RFC5 and VEGF expression levels are directly correlated. Our results further confirm this correlation as better VEGF silencing by the polyplex results in superior suppression of RFC5. The role of UBAP2 (ubiquitin associated protein 2) in cancer remains inconclusive as some reports have suggested a tumor suppressor role in hepatocellular cancer [51], while other studies on pancreatic cancer and glioblastoma have reported an oncogenic role for this gene [52].

The overall picture of pathways modulated by the polyplex treatment in A549 cells shows the regulation of a number of genes involved in cancer signaling (Figure 14). PVI-mediated siRNA delivery has a significant influence in the expression of several gene targets involved in cancer cell signaling when compared with free siRNA treatment (Figure 14). This could be attributed to the better internalization and endosomal escape evaluated by the polyplex. The in vitro studies indicate that VEGF silencing through the PVI polyplex has a positive influence in inhibiting the progression of lung cancer.

## Conclusion

This work has demonstrated the capability of poly(1-vinylimidazole) to serve as an efficient carrier of siRNA for gene silencing. The effectiveness of this carrier is due to its better cellular internalization as well as its ability to escape the endosome. The silencing of VEGF resulted in altered expression levels of genes responsible for proliferation and metastasis of lung cancer cells as evidenced by the microarray analysis. Also, VEGF silencing resulted in enhanced cytotoxicity of the chemotherapeutic agent 5-fluorouracil suggesting the promise of this strategy to be employed as an adjuvant therapy against lung cancer. The absence of cytotoxicity of the blank PVI polymer suggests that this carrier could be a cyto-compatible system for gene therapy.
